# Phytochemical Screening and Biological Activity of Female and Male Cones from *Pinus nigra* subsp. *laricio* (Poir.) Maire

**DOI:** 10.3390/antiox14111368

**Published:** 2025-11-18

**Authors:** Mary Fucile, Carmine Lupia, Martina Armentano, Mariangela Marrelli, Ekaterina Kozuharova, Giancarlo Statti, Filomena Conforti

**Affiliations:** 1 Department of Pharmacy, Health and Nutritional Sciences, University of Calabria, 87036 Rende, Italy; mary.fucile@unical.it (M.F.); martina.armentano@libero.it (M.A.); mariangela.marrelli@unical.it (M.M.); giancarlo.statti@unical.it (G.S.); 2 Mediterranean Ethnobotanical Conservatory, Sersale, 88054 Catanzaro, Italy; studiolupiacarmine@libero.it; 3 National Ethnobotanical Conservatory, Castelluccio Superiore, 85040 Potenza, Italy; 4 Department of Pharmacognosy, Faculty of Pharmacy, Medical University of Sofia, Dunav 2, 1000 Sofia, Bulgaria; inakozuharova@yahoo.co.uk

**Keywords:** antioxidant, polyphenols, flavonoid, anti-inflammatory, lipase, amylase, apolar compounds, obesity, diabetes, ethnobotany

## Abstract

The Corsican pine (*Pinus nigra* subsp. *laricio* (Poir.) Maire), a subspecies of black pine endemic to southern Italy, is widely known for the quality of its valuable timber, and the parts of the plant that are not used for this purpose are considered unusable production waste. In this study, we investigated the phytochemical profile and a series of biological activities of extracts from the female and male pine cones. The extracts were prepared by maceration with ethanol and subsequently fractionated using liquid-liquid separation. The total phenolic and flavonoid content, antioxidant potential (DPPH and β-carotene bleaching tests), anti-inflammatory activity (nitric oxide inhibition in RAW 264.7 cells), and enzymatic inhibition against pancreatic lipase and α-amylase were determined. The female cones showed a higher crude extract yield and total phenolic content (76.4 mg GAE/g) than the male cones, while the latter were richer in flavonoids. The extracts from the female cones showed higher antioxidant and pancreatic lipase inhibitory activities. On the contrary, extracts from male cones showed greater activity against α-amylase, with the dichloromethane fraction proving to be the most potent (IC_50_ = 35.28 ± 3.08 µg/mL). The hexane fraction of female cones also showed significant anti-inflammatory activity (IC_50_ = 107.50 ± 15.22 µg/mL). Our results reveal that the pine cones of *Pinus nigra* subsp. *laricio* (Poir.) Maire are a rich source of bioactive compounds. These results provide the first scientific evidence of the potential of extracts from this still poorly studied part of the plant for further investigation of their antioxidant and anti-inflammatory capabilities.

## 1. Introduction

Research into the therapeutic potential of natural products has grown significantly in recent years, particularly in the identification of compounds that may have antioxidant and anti-inflammatory effects [1], as well as antimicrobial and anticancer effects [2]. This rediscovery of natural extracts stems from increased demand from the industry market for the production of nutraceutical products [3], as well as the need for new molecules that have cytotoxic activity on cancer cells [4] and new molecules that can help in the ever-growing phenomenon of antibiotic resistance [5]. The exploration of natural bioactive compounds has marked a turning point in the biomedical sector in recent decades. This growing interest is fueled by the increasing demand for new biopharmaceutical products and functional foods, which has stimulated extensive research on various plant species as sources of secondary metabolites useful in supporting traditional medicine [6]. Biomolecules derived from plant species show a growing correlation between traditional ethnopharmacological uses and scientifically validated pharmacological activities [7]. In the Sila region (Calabria, Italy), the black pine was traditionally used by local populations for various purposes: for example, the resin was used as a painkiller, and the buds were used in the form of a decoction as a cough remedy and for bronchial diseases [8]. The young female cones of *Pinus* sp. div. (*P. nigra* J. F. Arnold and *P. sylvestris* L.) in the form of syrup are the plant substance reported together with 26 others in the Central Rhodopes of Bulgaria used traditionally to treat respiratory problems [9,10]. Similarly, the young shoots and female cones of *Abies alba* Mill., *Picea abies* (L.) H. Karst., *Pinus nigra*, and *Pinus sylvestris* are used for syrup production and applied traditionally to treat respiratory diseases in Transylvania [11]. In Serbia the syrup is made from *Pinus nigra* shoots and again is used traditionally to treat pulmonal diseases. In addition, there a recipe quoting that two tablespoons of the black pine (*Pinus nigra*) pollen, locally known as “flower powder” (in Serbian, “cvetni prah”) is mixed with bee-honey to prepare a remedy for respiratory ailments [12]. The thick syrup with a honey-like consistency, obtained after prolonged boiling with sugar in Bulgaria is called “pine honey”. Interestingly “pine honey” in the neighboring Turkey and Greece is a different product. It is produced by honeybees collecting the honeydew of *Marchalina hellenica (Gennadius)* that suck the sap *Pinus brutia* Ten. and *Pinus halepensis* Mill. [13,14,15] with notable antioxidant activity as well as antiproliferative, anticholinesterase, anti-inflammatory, tyrosinase inhibitory, and urease inhibitory effects related to the phenolic content [16]. Turkey is the leading producer of pine honey worldwide, accounting for 90% of global production [17]. The knowledge of these traditional practices could provide an important empirical basis for screening new bioactive molecules, allowing research to be directed towards secondary metabolites traditionally documented for their applications. Their scientific validation, through their identification and analysis of their molecular targets, could lead to the discovery of new therapeutic agents. Among various sources, the *Pinus* genus stands out for its richness in secondary metabolites known for their various biological activities. This makes it a subject of particular interest for scientific research in the biomedical field. Several recent scientific studies have shown that pine cone extracts are a rich source of biologically active compounds, making them a potential material in various commercial sectors [18,19,20]. Their chemical composition is like that of coniferous wood, consisting mainly of cellulose, lignin, and hemicellulose. Beyond these structural components, pine cone extracts contain various classes of molecules, including terpenoids, such as oleoresins [21], which act as a natural defense mechanism. Pine extracts have been extensively studied and are characterized by the presence of terpenic substances such as monoterpenes and sesquiterpenes, including pinene, camphene, and limonene [22]. However, what makes them interesting from a scientific and application point of view is their high concentration of phenolic compounds. Previous studies have highlighted their rich phytochemical profile, revealing the presence of polyphenols, flavonoids, and terpenes, which are known for their beneficial effects on health [23,24,25]. Furthermore, the presence of phenolic compounds, such as phenolic acids, flavonols, and flavonoids such as catechins, further enriches the therapeutic potential of pine extracts, giving them antioxidant and cytoprotective properties [26,27,28]. These polar compounds have been the subject of numerous recent studies that have explored their therapeutic effects and mechanisms of action on human health. Species belonging to the Pinus genus represent a significant reservoir of secondary metabolites, particularly phenolic compounds and tannins, whose composition is influenced by the part of the plant and the extraction method [29]. Recent research has validated the functional potential of these extracts. For example, the polar fraction of the bark of *P. pinaster* Ait. and *P. pinea* L. exhibits powerful antioxidant activity, with free radical scavenging efficacy (DPPH and ABTS) comparable to that of known standards. Significant bactericidal action against clinically relevant pathogens such as *Staphylococcus aureus* and *Escherichia coli* has also been documented. The study on *P. pinea* L., in particular, revealed a greater abundance of total polyphenols, leading to the identification of compounds such as quinic acid and protocatechuic acid in this specific matrix [29]. Despite solid evidence of their functional properties and potential application in the nutraceutical, cosmetic, and pharmaceutical sectors, the full utilization of pine residues remains underdeveloped [30]. The variation in the concentrations of individual bioactive compounds, although the presence of polyphenols is universally confirmed, makes it crucial to optimize extraction protocols to ensure the sustainability of the process and maximize functional yield. The adoption of a circular economy model represents a key strategy for sustainability, emphasizing the bio-valorization of agroforestry by-products as renewable alternatives to finite resources. In this context, residues generated from the processing of forest products, traditionally considered waste streams, are recognized as promising matrices for the extraction of high-value compounds [31,32].

Following on from a previous investigation in which extracts from apical shoots and branches of *Pinus nigra subsp. laricio* (Poir.) Maire were analyzed, highlighting their antioxidant, anti-inflammatory, and enzyme-inhibiting properties. Building on this research, the present study aims for the first time to evaluate the antioxidant, anti-inflammatory, and enzyme-inhibiting activities of ethanol extracts derived from male (pollen cones or microsporophylls) and female cones (ovule cones or macrosporophylls) of *Pinus nigra* subsp. *laricio* (Poir.) Maire. This investigation was conducted using established in vitro tests, including the DPPH and beta-carotene bleaching tests for antioxidant capacity, inhibition of nitric oxide production in murine RAW 264.7 macrophages for anti-inflammatory potential, and tests targeting pancreatic lipase and alpha-amylase for inhibitory effects on enzymes involved in lipid and carbohydrate absorption.

## 2. Materials and Methods

### 2.1. Reagents and Solvents

All reagents and solvents used in this study, including Folin–Ciocalteu reagent, aluminum chloride, sodium carbonate, 2,2-diphenyl-1-picrylhydrazyl (DPPH), ascorbic acid, Griess reagent, 3-(4,5-dimethylthiazol-2-yl)-2,5-diphenyltetrazolium bromide (MTT), Dulbecco’s modified Eagle’s medium (DMEM), l-glutamine, fetal bovine serum (FBS), antibiotic/antifungal solution (penicillin/streptomycin), lipopolysaccharide (LPS), indomethacin, type II lipase from porcine pancreas, 4-nitrophenyl caprylate (p-NPC), TRIZMA base, orlistat, pig pancreas α-amylase, potato starch, sodium and potassium tartrate, sodium hydroxide, 3,5-dinitrosalicylic acid, phosphate-buffered saline (PBS), and acarbose were purchased from Sigma-Aldrich S.p.A. (Milan, Italy). Solvents were supplied by VWR International s.r.l. (Milan, Italy).

### 2.2. Plant Material and Sample Preparation

The samples were collected in the Sila National Park (Calabria, Italy) in June. An herbarium sample (leg. det. Carmine Lupia) was deposited at Mediterranean Etnobotanical Conservatory, Sersale, Catanzaro (position number 22 of the Pinaceae section). Both samples were used fresh. The female pine cones (1382.20 g) were cut into smaller pieces, while the male pine cones (399.10 g) were used whole. The difference in the preparation protocol is due to the marked morphological diversity of male cones, which are significantly thinner and more delicate. The plant material from both parts of the plant was macerated in ethanol (for 48 h, repeated 3 times) at room temperature, maintaining a plant/solvent ratio of 1:8 (g/mL). The total extracts were filtered and concentrated under reduced pressure. A portion of the resulting crude extract was dissolved in 100 mL of a methanol/water mixture in a 9:1 ratio. For fractionation, a liquid-liquid partitioning technique was used with an equal volume of *n*-hexane, continuing the operation until complete exhaustion. The unexhausted portion of the extract was dried, redissolved in 100 mL of water, and fractionated again using the same partitioning methodology. Dichloromethane (CH_2_Cl_2_) and then ethyl acetate (AcOEt) were used in sequence. The residual aqueous fraction (H_2_O) was also subsequently included in the analysis. The yield percentages of all extracts and fractions obtained were determined by calculating them relative to the initial mass of fresh plant material. Finally, the low polarity fractions (n-hexane and dichloromethane) were examined by gas chromatography coupled with mass spectrometry (GC-MS).

### 2.3. GC-MS Analysis of Apolar Fractions

Gas chromatography coupled with mass spectrometry (GC-MS) analysis was performed on the n-hexane and dichloromethane fractions obtained from each sample. For this purpose, a Hewlett-Packard model 6890 chromatograph was used, connected to a Hewlett-Packard model 5973 selective mass spectrometry detector. Samples (1 µL) were introduced using a 10 µL Hamilton microsyringe. The instrument was equipped with a 100% dimethylpolysiloxane SE-30 capillary column (length: 30 m, inner diameter: 0.25 mm, film thickness: 0.25 μm). Helium was used as the carrier gas. The column’s temperature program involved an increase from 60 °C at a rate of 16 °C, resulting in a total elution time of 20 min. The injector temperature was established at 250 °C. Helium’s linear velocity was maintained at 0.00167 cm/s. The operating parameters for the mass spectrometer were set as follows: ion source energy: 70 eV; ion source temperature: 230 °C; electron current: 34.6 μA; vacuum: 10^−5^ torr. Mass spectra were acquired over a mass-to-charge range (*m*/*z*) from 40 to 800 amu, with a scanning rate of 1 scan/s. Compounds were tentatively identified by comparing the obtained spectra with those available in the Wiley mass spectral library integrated within the GC-MS system.

### 2.4. Total Phenolic and Flavonoid Content

The phenol and flavonoid content was determined using the methods described above [33]. The total phenolic content was determined for the raw extracts using the Folin–Ciocalteu reagent. 1 mL of Folin–Ciocalteu reagent was mixed with 1 mL of Na_2_CO_3_ (7.5% *w*/*v*) to 200 µL of samples at a concentration of 2 mg/mL, dissolved in a solution prepared with acetone/methanol/water/acetic acid (40:40:20:0.1) after incubation for 1 h at 60 °C. The absorbance was recorded at 726 nm two hours following the initial reaction. The flavonoid content was determined through the addition of 2% AlCl_3_ solution to 1 mL of sample (concentration: 2 mg/mL) prepared in 80% EtOH. After 15 min, the absorbance of the resulting complex was measured at 430 nm. The calibration curves of the standards—specifically chlorogenic acid for polyphenols and quercetin for flavonoids—were used to interpolate the measured values. The final results are presented as milligram equivalents per gram (mg equivalents/g) of fresh plant material. All analyses were performed in triplicate and the results are expressed as mean ± SE.

### 2.5. Antioxidant Activity

#### 2.5.1. Determination of Free Radical Scavenging Activity (DPPH Test)

To study the antiradical activity of the extracts, the 2,2-diphenyl-1-picrylhydrazyl (DPPH) test described by Ceramella et al. was performed with some modifications [34]. All samples were diluted in series to final concentrations ranging from 2.5 to 1000 µg/mL, and 200 µL of each was added to 800 µL of a 0.1 mM DPPH methanolic solution. Ascorbic acid was used as a positive control. After 30 min of incubation in the dark, the absorbance was measured at 517 nm.

#### 2.5.2. Beta-Carotene Bleaching Test

The test is based on the beta-carotene discoloration induced by oxidation, which occurs because of interaction with the thermo-degradation products of linoleic acid [35]. One milliliter of this solution was then mixed with 60 µL of linoleic acid and 600 µL of Tween 20. The mixture was initially concentrated using a rotary evaporator to ensure the complete removal of chloroform. Subsequently, it was diluted with 200 mL of distilled water and vigorously agitated to produce a uniform, homogeneous emulsion. For the assay, 5 mL aliquots of this emulsion were dispensed into test tubes. Each tube contained 200 µL of the extracts and fractions at various concentrations, ranging from 1 to 100 µg/mL. The test tubes were gently mixed and immediately transferred for incubation in a thermostatic bath set at 45 °C for 60 min. Propyl gallate served as the positive control for this experiment. A blank sample, which consisted solely of the emulsion without β-carotene, was utilized as the analytical reference. The absorbance of the samples, along with the positive and negative controls, was read at 470 nm. Measurements were taken at three time points: the initial time (t = 0), after 30 min of incubation, and finally after 60 min. All analyses were conducted in triplicate, and the reported values represent the calculated averages of these measurements.

### 2.6. In Vitro Evaluation of Nitric Oxide (NO) Production Inhibition and Cytotoxicity

The RAW 264.7 murine macrophage cell line was employed to assess the impact of the extracts on LPS-induced nitric oxide (NO) production inhibition. Cells were maintained at 37 °C in an atmosphere containing 5% CO_2_, utilizing culture flasks filled with DMEM medium. This medium was enhanced with 1% L-glutamine, 10% fetal bovine serum (FBS), and 1% antibiotic/antifungal solution (penicillin/streptomycin). Upon reaching confluence, the cells were detached by scraping, collected through centrifugation (1500 rpm for 10 min), and subsequently resuspended in fresh medium. Cell viability and cell count were confirmed using the standard trypan blue exclusion method. The cells were then seeded into 96-well plates (at a density of 1 × 10^4^ cells/well and utilized for experiments after achieving confluence (24 h later). Nitrite production in the cell culture supernatant was quantified 24 h post-treatment with various extract concentrations, ranging from 6.25 to 1000 µg/mL, using the Griess reagent. Specifically, 100 µL of the supernatant was combined with 100 µL of the reagent in a 96-well plate, and the resulting absorbance was recorded at 550 nm using a microplate reader (GDV DV 990 B/V, Rome, Italy) [36]. At the same time, cytotoxicity was assessed using the MTT assay to confirm the absence of toxic effects of the extracts on RAW 264.7 macrophages. At the end of the experiment, 100 µL/well of a 0.5 mg/mL MTT solution was added to the cells. After four hours, the supernatant was removed and the formazan crystals formed were dissolved by adding 100 µL/well of dimethyl sulfoxide (DMSO). The absorbance was then measured at 570 nm.

### 2.7. Pancreatic Lipase Inhibition Assay

To determine the extract’s inhibitory capability against pancreatic lipase, the substrate utilized was p-nitrophenyl caprylate (NPC) [37]. A 1 g/L aqueous solution of porcine pancreatic lipase (Type II) was first prepared. Separately, a 5 mmol/L} solution of 4-nitrophenyl octanoate (NPC) was prepared using dimethyl sulfoxide (DMSO). The reaction mixture was assembled in a test tube by combining: 100 µL of NPC (5 mmol/L); 4 mL of Tris-HCl buffer (pH = 8); 100 µL of the extract/fraction (concentrations ranging from 0.0625 to 2.5 mg/mL); 100 µL of the enzyme solution. This mixture was then pre-incubated at 37 °C for 25 min before the substrate was added to initiate the reaction. In the control group, the extract was replaced with an equivalent volume of dimethyl sulfoxide (DMSO). Orlistat, at a final concentration of 20 µg/mL served as the positive control. The reaction’s absorbance was measured at 412 nm. For each extract, a blank measurement without the enzyme was also taken. This was carried out to account for and eliminate any potential interference in the final reading caused by the inherent color of the extract itself.

### 2.8. Alpha-Amylase Inhibition Assay

The inhibition of pancreatic α-amylase was evaluated using the 3,5-dinitrosalicylic acid (DNSA) chromogenic method as previously described [38]. A 0.5% (*w*/*v*) potato starch solution was prepared in a sodium phosphate buffer (0.02 mM) with sodium chloride (6.7 mM) at a pH of 7. In a test tube, 250 µL of the extract/fraction (7.8125–250 µg/mL) and 250 µL of α-amylase solution (0.5 mg/mL) were combined. The mixture was incubated at 37 °C for 10 min. Next, 250 µL of starch solution (0.5% *w*/*v*) was added, and incubation continued for another 15 min. To stop the reaction, 2 mL of DNSA reagent was added, and the test tube was incubated at 85 °C for 15 min. The reaction was then cooled and diluted with 2 mL of distilled water. The absorbance was measured at a wavelength of 540 nm. For each extract, a blank sample without enzyme was measured. Acarbose was used as a positive control, while another control was prepared by replacing the extract with the solvent alone. All experiments were performed in triplicate.

## 3. Results

### 3.1. Yields of Extraction and Phytochemical Profile

**Figure 1:**
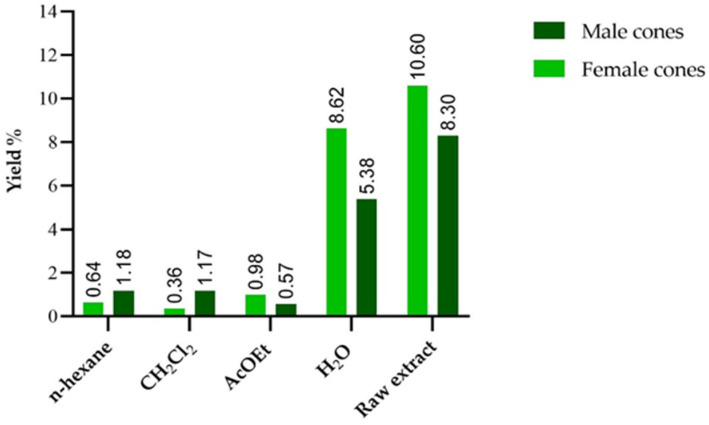
Extraction yields of the two extracts. The % are expressed on g of fresh plant material.

The samples were prepared by maceration using ethanol as a solvent. The extraction yields in percentage are shown in Table 1. Female cones show a higher extraction yield than male cones for the crude extract (10.60% and 8.30%, respectively). The differences in fraction yields, expressed in Figure 1, suggest a different chemical composition between the two types of pine cones. Male cones appear to be richer in medium- and low-polarity compounds, comparing the yields obtained with n-hexane and dichloromethane, while the yields of female cones suggest a higher content of more polar compounds. For both types of pine cones, a large part of the compounds present in the crude extract was not extracted by the organic solvents used. This results in a high yield of the aqueous residue, which is the most abundant fraction.

Female cones showed a significantly higher content of total phenolic compounds than male cones, while the concentration of flavonoid compounds was higher in male cones.

Preliminary analysis on the apolar fractions of the two extracts using gas chromatography shows that female cones are mainly composed of fatty acids and hydrocarbons, while male cones contain a variety of volatile compounds such as aldehydes, ketones, and terpenoids. Only a few compounds are present in both extracts, such as palmitic acid, α-linolenic acid and 1-Octadecene (Table 2 and Table 3 and Figure S1). Alpha-linolenic acid has a higher relative area percentage in male cones (2.2% of the total peak areas in the total ion current (TIC)) than in female cones (0.6%), while oleic acid (3.7%) is only present in female cones and is not found in male cones. Fragrant compounds, such as fenchyl acetate and 2-phenylethyl isovalerate, were also detected in female cones, albeit in small quantities.

### 3.2. Evaluation of Antioxidant Potential

**Figure 2:**
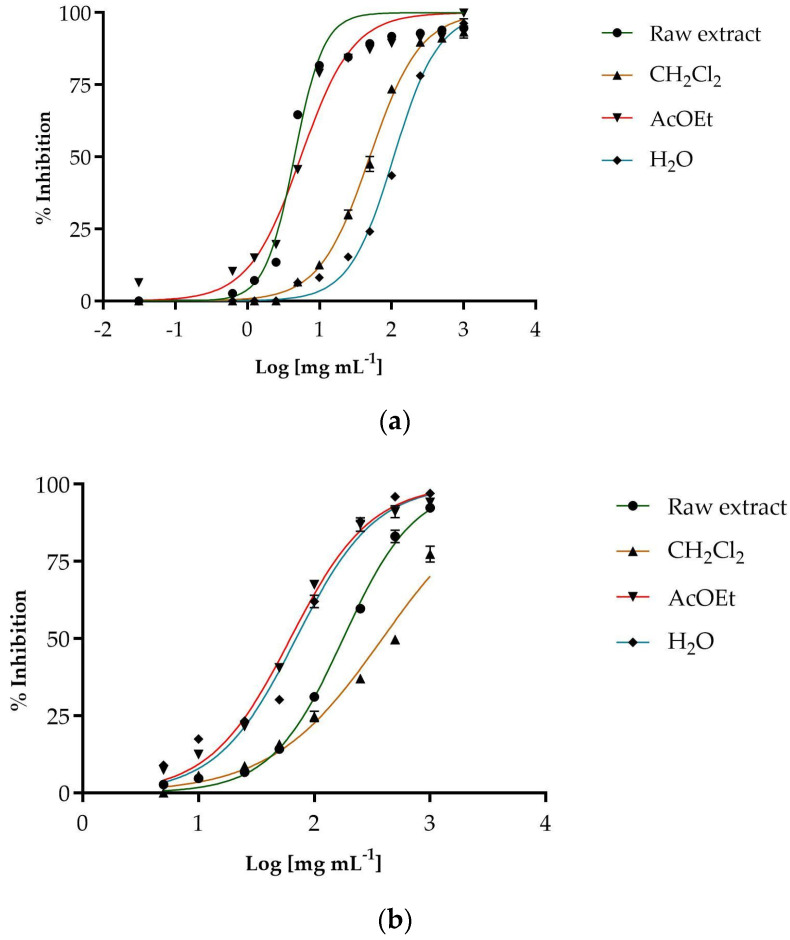
Antiradical activity of the female cones extracts (**a**) and male cones extract (**b**).

The antioxidant potential of the extracts examined was evaluated using two colorimetric tests: the DPPH test for free radical scavenging activity and the β-carotene bleaching test to evaluate the protective effect against linoleic acid oxidation. The data clearly show that female cones have much higher antioxidant activity than male cones (Table 4). This applies to both the total extract and the individual fractions. The best antiradical scavenging activity was shown by the female cones (Figure 2).

**Table 4:** Antioxidant activity of the crude extracts and their derived fractions from female and male cones, as measured by DPPH and β-carotene bleaching test.

Sample	Fraction	DPPH Test	β-Carotene Bleaching Test	
		IC_50_ (µg/mL)		
			30 min	60 min
Female cones	Raw extract	4.50 ± 0.10 ^a^	3.03 ± 0.06 ^b^	10.77 ± 0.37 ^c^
	*n*-hexane	>1000	17.89 ± 0.39 ^g^	49.72 ± 2.42 ^f^
	CH_2_Cl_2_	49.80 ± 2.41 ^b^	4.56 ± 0.13 ^c^	16.24 ± 0.64 ^d^
	AcOEt	5.52 ± 0.08 ^a^	4.44 ± 0.18 ^c^	18.21 ± 0.16 ^d^
	H_2_ O	107.17 ± 1.94 ^d^	9.57 ± 0.23 ^e^	40.24 ± 1.13 ^e^
Male cones	Raw extract	178.03±1.29 ^e^	15.47 ± 0.27 ^f^	>100
	*n*-hexane	>1000	>100	>100
	CH_2_Cl_2_	389.70 ± 12.35 ^f^	>100	>100
	AcOEt	62.33 ± 0.50 ^c^	5.09 ± 0.08 ^d^	8.27 ± 0.10 ^b^
	H_2_O	69.88 ± 1.09 ^c^	>100	>100
Ascorbic acid *		2.00 ± 0.01 ^a^	-	-
Propil gallate *		-	1.00 ± 0.02 ^a^	1.00 ± 0.02 ^a^

Data expressed as mean ± SEM (*n* = 3). * Positive controls. Different letters along column indicate statistically significant differences at *p* < 0.05 (Bonferroni post hoc test).

The fraction with the strongest antioxidant activity in female cones is the ethyl acetate fraction (IC_50_ = 5.52 ± 0.08 µg/mL). In male cones, the most active fraction is the ethyl acetate fraction (IC_50_ = 62.33 ± 0.50 µg/mL), but its activity is far lower than that shown by the same fraction in female cones. The n-hexane fractions of both cones showed no activity against free radicals at the concentrations tested.

The results of the β-carotene discoloration test also favor female cones, which show a much stronger antioxidant effect after 30 and 60 min of incubation at 45 °C. In addition to the raw extract showing the best activity, the fractions of female cones also demonstrate good protection against peroxidation, with the ethyl acetate fraction being particularly effective. The fractions of the male cones, on the other hand, show limited or no activity, except for the ethyl acetate fraction, which shows an excellent ability to inhibit lipid peroxidation, even after 60 min of incubation, even better than the same fraction of the female cones, with an IC_50_ value of 8.27± 0.10 µg/mL.

### 3.3. Quantification of Nitric Oxide Production

Interest in researching antioxidants derived from natural products is constantly growing, given that consuming antioxidants can have multiple health benefits [39]. When activated by the immune system, macrophages produce large amounts of reactive oxygen species and reactive nitrogen species. Their prolonged activation leads to epigenetic modifications, contributing to the development of chronic diseases [40]. Analysis of the data, shown in Table 5, revealed significant differences between the raw extracts and their fractions. The raw extract from male cones showed no cytotoxic effects at the concentrations tested, while the raw extract from female cones showed cytotoxicity at high concentrations, with an IC_50_ of 969.10 ± 10.78 µg/mL. The fractions, except for the hexane fraction of the female cones, showed toxicity IC_50_ values comparable to the effective concentrations. The only fraction worth mentioning is the n-hexane fraction of female cones, which is not only more effective (IC_50_ = 107.50 ± 15.22 µg/mL) but also shows a significantly better safety margin than its crude extract.

### 3.4. Pancreatic Lipase Inhibition Activity

**Figure 3:**
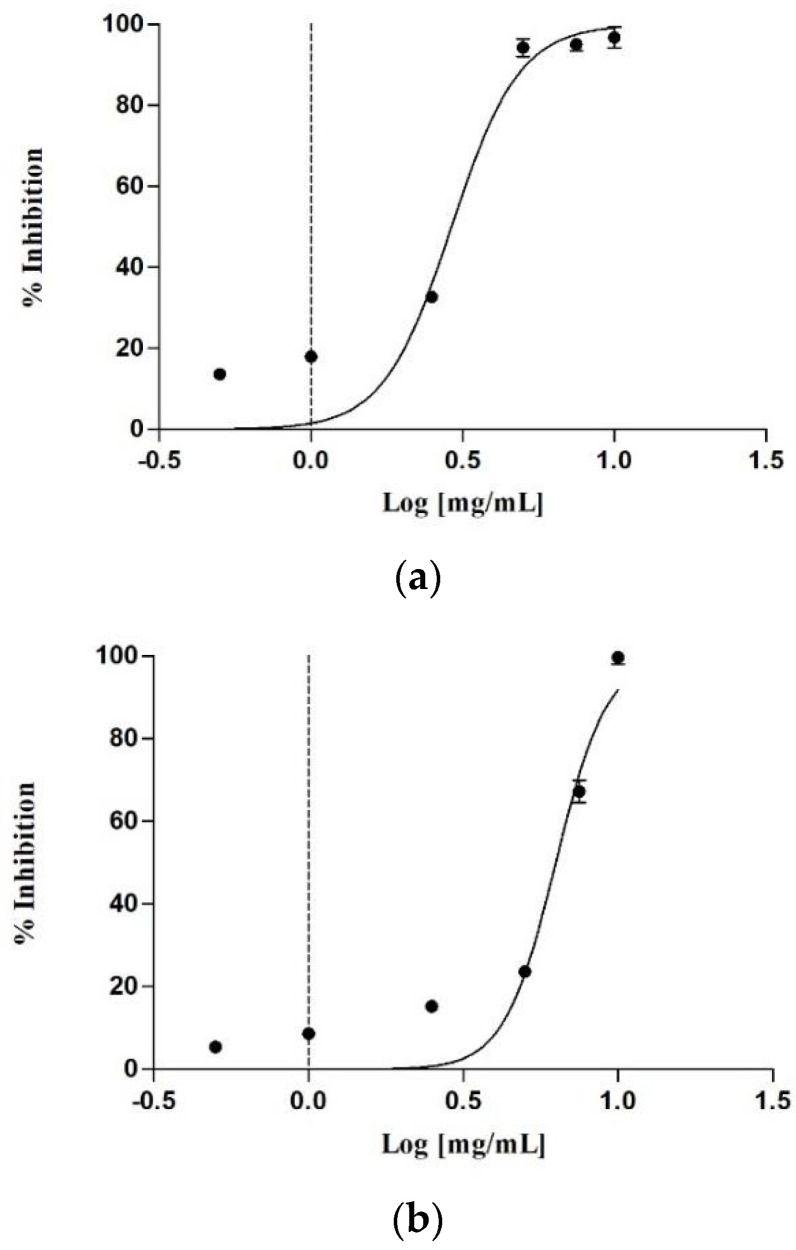
Concentration-dependent lipase inhibitory activity of the raw extract of the female cones (**a**) and male cones (**b**).

Obesity is a complex, multifactorial condition that contributes significantly to various comorbidities, including type 2 diabetes, cardiovascular disease, and certain forms of cancer. To date, pharmacological strategies for treating obesity have mainly focused on reducing food intake, increasing energy expenditure, or decreasing nutrient absorption [41]. Among the most effective therapeutic targets for reducing nutrient absorption, pancreatic lipase inhibition is a well-established and clinically validated approach [42]. For this purpose, the inhibitory activity against the enzyme was evaluated by monitoring the hydrolysis of a chromogenic substrate, p-nitrophenyl caprylate (p-NPC). The reaction was measured spectrophotometrically by detecting the absorbance of the reaction product. The analysis was limited to total extracts and their aqueous fractions, due to the low yields of the other fractions. As shown in Table 6, the raw extract from female cones exhibited the best enzyme inhibition, with an IC_50_ of 0.20 ± 0.01 mg/mL. This was followed by the male cones, which had an IC_50_ of 0.52 ± 0.01 mg/mL. Both aqueous fractions also showed good activity, though at higher concentrations. Figure 3 illustrates the dose-dependent inhibition of the raw extract. In particular, the female cone extract inhibited 21.49 ± 0.13% of the activity at the lowest concentration tested (0.03125 mg/mL) and 71.93 ± 0.91% at 1 mg/mL. In contrast, the male cone extract showed no activity at the lowest concentration, with an enzyme inhibition of 53.69 ± 0.26% at 1 mg/mL.

### 3.5. Evaluation of α-Amylase Inhibitory Activity

Starch is the main source of carbohydrates and represents a significant part of the energy intake in the human diet. Consistently high blood glucose levels can damage tissues and organs, leading to serious complications typical of the so-called Metabolic Syndrome and its progression, including vascular disease, neuropathy, retinopathy, and kidney problems [43,44]. As can be seen from Table 7, this time, the male cones showed better activity. The raw extract and all fractions of the male cones showed activity at the concentrations tested. In contrast, the female cone samples, except for the crude extract, showed no inhibition or showed inhibition only at the highest concentrations. Specifically, the most effective fraction was the dichloromethane fraction from the male cones (IC_50_ = 35.28 ± 3.08 µg/mL), followed by the aqueous residue, also from the male cones (IC_50_ = 46.77 ± 3.15 µg/mL).

## 4. Discussion

The data obtained indicate that extracts from both female and male cones of *Pinus nigra* subsp. *laricio* (Poir.) Maire possess biological activity in vitro, particularly with regard to their antioxidant potential and their ability to inhibit key enzymes linked to metabolic disorders. The ecological and economic importance of pine forests in Europe, which cover some of the largest areas on the continent, generates significant quantities of waste from timber processing. The use of these industrial by-products is increasingly becoming a strategy in line with the principles of the circular economy, which aims to transform waste into resources. Several recent studies have explored alternative uses for pine by-products in various fields: from the production of eco-sustainable timber [45] to the selective recovery of metals [46], as well as uses in the pharmaceutical, cosmetic, and nutraceutical fields [47]. Considering that the larch pine has been used since ancient Roman times for its valuable wood [10], the use of processing waste may be useful in a circular economy context. Although female cones have a higher total extract yield, examination of the fractions indicates a distinct distribution of metabolites. Male cones, with higher yields in the medium- and low-polarity fractions (n-hexane and dichloromethane), suggest a greater abundance of lipophilic compounds, in line with the identification of fatty acids, aldehydes, ketones, and terpenoids. In contrast, female cones produced a significantly higher yield in the aqueous fraction and ethyl acetate, indicating a higher concentration of compounds with higher polarity. This compositional diversity is consistent with data on phenolic compounds and flavonoids: female cones are significantly richer in total polyphenols, while male cones contain a higher concentration of total flavonoids. Our quantitative data confirm and expand on this trend, highlighting that female cones have an extremely high total phenolic content (76.4 ± 1.80 mg GAE/g) compared to male cones (18.2 ± 0.60 mg GAE/g) and other parts of the plant previously studied (apical shoots: 20.66 ± 0.27 mg GAE/g; branches: 18.10 ± 0.70 mg GAE/g). Conversely, total flavonoid content is highest in male cones (1.02 ± 0.03 mg QE/g) compared to all other parts of the plant considered [38]. A study carried out on six different conifers taxa (*Cedrus atlantica*, *Larix decidua*, *Picea abies*, *Pinus nigra*, *Pseudotsuga menziesii*, *Tsuga canadensis*) showed that the content of phenolic compounds decreases with the degree of cone maturation [48]. In general, the phenolic compound content in plant tissues is influenced by various environmental and genetic factors, as well as by the degree of tissue ripeness. During the fruit ripening process, for example, there is a decrease in phenolic acids in favor of anthocyanins [49]. The antioxidant activity generally shows greater efficacy in female cone extracts than in male cone extracts. The crude extract of female cones had a significantly lower IC_50_ value (4.50 ± 0.10 µg/mL) for the DPPH test than that of male cones. These values place the antioxidant activity of extracts from female cones of *P. nigra* subsp. *laricio* in a range comparable to other conifer taxa. For example, studies conducted on 80% acetone extracts of cones from different conifers reported significantly varying IC_50_ values in the DPPH test: from 4.42 µg/mL (mature cones of *Metasequoia glyptostroboides*) to 7.83 µg/mL (green cones of *Tsuga canadensis*) [50]. A noteworthy exception occurred in the inhibition of lipid peroxidation: although the male crude extract showed lower efficacy than the female extract, its ethyl acetate fraction showed a better IC_50_ value than the same fraction of the female cone. This data suggests the enrichment of specific antioxidant compounds in this fraction, the nature of which requires further chemical investigation. The enhanced activity could be attributed to the presence of flavonoids, compounds that tend to concentrate in the ethyl acetate fraction [51] and are present in higher concentrations in male cones

The inhibitory activity against pancreatic lipase was higher in extracts from female cones, which is consistent with their higher total polyphenol content. It is hypothesized that this activity is partly mediated by phenolic compounds, including flavonoids, in line with molecular docking studies in the literature that indicate their ability to bind to the active site of the enzyme [37]. The difference in activity between male and female cones is probably determined by the different distribution of bioactive compounds in the two types of cones.

As regards activity against the α-amylase enzyme, male cones showed greater activity, with more favorable IC_50_ values for the dichloromethane fraction and the aqueous residue. These data suggest that inhibition is mediated by heterogeneous compounds with a wide range of polarity. The increased activity of the dichloromethane fraction could suggest the presence of compounds with medium lipophilicity, such as non-glycosylated phenolic acids. This hypothesis is consistent with studies that demonstrated the inhibitory effect of phenolic acids on α-amylase, highlighting how the presence of specific hydroxyl groups in position 2 on the benzene ring drastically increases their inhibitory efficacy [52]. Furthermore, the higher flavonoid content in male cones suggests that these compounds may contribute significantly to the overall activity [53]. This hypothesis could be supported by molecular docking studies, which have shown that flavonoids can effectively bind to the active site of enzymes involved in carbohydrate metabolism, such as α-amylase [54]. Regarding the anti-inflammatory potential of the *Pinus nigra* cone extracts examined, these results show promising activity in inhibiting nitric oxide in female cones, particularly for the less polar fractions. This is in line with previous study [38], conducted on extracts from *Pinus nigra* branches and shoots, which showed that non-polar fractions (hexane and dichloromethane) are particularly effective in inhibiting NO production in RAW 264.7 macrophages, with IC_50_ values comparable to the positive controls used for the n-hexane (IC_50_ = 43.52 ± 2.34 µg/mL) and dichloromethane (IC_50_ = 50.68 ± 3.63 µg/mL) fractions of the branch extract. In line with the anti-inflammatory potential exhibited by extracts obtained from the same species, another study conducted on hydroalcoholic extracts of *Pinus densiflora* needles [55] showed that they can suppress acute inflammation. Jeong, S. Y. and colleagues (2022) reported a significant reduction in NO, with IC_50_ values of 27.44 µg/mL, and a reduction in pro-inflammatory mediators such as tumor necrosis factor-α (TNF-α) and interleukin-1β (IL-1β) using the same macrophage cell line (RAW 264.7). However, the researchers attributed these effects to more polar compounds, particularly the presence of flavonoids such as taxifolin and quercetin glucoside. The effectiveness of less polar fractions presented in this study could therefore be linked to their higher content of lipophilic compounds, such as specific fatty acids identified in pine cones. For example, oleic acid, an important monounsaturated fatty acid often found in plant tissues, is well documented for its anti-inflammatory effects through mechanisms such as the inhibition of pro-inflammatory cytokines, the regulation of cell membrane fluidity, and the activation of natural activator of sirtuin 1 (SIRT1) [56]. In addition, α-linolenic acid has also demonstrated significant anti-inflammatory potential attributed to the inhibition of key enzymes such as cyclooxygenase (COX-1, COX-2) [57]. This study provides for the first time basic data on the potential biological activities of female and male cones of *Pinus nigra* subsp. *laricio* (Poir.) Maire. This study adds basic data on the properties of a species that has been little studied from a chemical-biological point of view. The use of these forest by-products is consistent with the principles of the circular economy. Future research should focus on characterizing the compounds, particularly those present in the most effective fractions for each activity, and explaining their specific mechanism of action.

## 5. Conclusions

The results of this study indicate that extracts from the cones of *Pinus nigra* subsp. *laricio* (Poir.) Maire show in vitro activity in inhibiting oxidative and inflammatory processes and key enzymes involved in metabolic disorders. These results are the first to document such effects for this plant material. Although further investigation is needed to identify the responsible compounds and molecular mechanisms involved, our data add basic information on the biological properties of extracts from a plant that has been little studied to date. The use of forest by-products, such as pine cones, is an approach that aligns with the principles of the circular economy. In summary, the in vitro results of this study justify further exploration of these extracts as a potential source of bioactive compounds. The activities observed provide a scientific basis for hypothesizing future preclinical and clinical studies aimed at evaluating their applicability in industrial and therapeutic contexts.

## Figures and Tables

**Table 1:** Total phenolic and flavonoid content.

Sample	TPC ^1^	TFC ^2^
Female cones	76.4 ± 1.80 ^a^	0.54 ± 0.03 ^b^
Male cones	18.2 ± 0.60 ^b^	1.02 ± 0.03 ^a^

^1^ Total phenolic content. ^2^ Total flavonoid content. Data are mean ± SE (*n* = 3). The results are expressed as mg equivalents of chlorogenic acid (TPC) or quercetin (TFC) per g of fresh plant material. Different letters within columns (TPC and TFC) indicate statistically significant differences (*p* < 0.05, Student’s *t*-test).

**Table 2:** Identification of apolar compounds found within the nonpolar fraction of the female cones.

N.	Compound ^(a)^	Rt ^(b)^	Mass Fragmentation (Mass *m*/*z* Values)	RAP ^(c)^
	***n*** **-hexane**			
1	α-Fenchyl acetate	11.26	196 (M^+^), 80, 95, 121, 136, 154	0.1
2	2-Phenylethyl isovalerate	13.05	206 (M^+^), 57, 91, 104	0.1
3	Palmitic acid	16.49	256 (M^+^), 57, 60, 73, 213	0.7
4	Cyclohexadecane	16.36	224 (M^+^), 55, 83, 97	1.5
5	Oleic acid	16.43	282 (M^+^), 41, 55, 73, 264	3.7
6	1-Octadecene	17.00	252 (M^+^), 55, 69, 97, 125	4.7
7	Linoleic acid, methyl ester	17.12	294 (M^+^), 67, 81, 95, 263	0.8
8	Ethyl linoleate	17.51	308 (M^+^), 67, 81, 220, 263	0.8
9	α-Linolenic acid	17.55	306 (M^+^), 55, 79, 95, 241, 261	0.6
10	1-phenanthrenecarboxaldehyde, 1,2,3,4,4a,9,10,10a-octahydro-1,4a-dimethyl-7(1-methylethyl)-1R-(1.α, 4a β, 10a.α)	18.33	284 (M^+^), 91, 159, 173, 241, 269	3.2
11	4-Epidehydroabietol	18.84	286 (M^+^), 159, 173, 253, 271	4.0
12	Kauren-19-ol	19.50	287 (M^+^), 81, 95, 121, 257, 271	1.5
	**Dichloromethane**			
1	2-Ethylhexyl-4-methoxycinnamate	18.48	290 (M^+^), 133, 161, 178	1.4
2	2,6,10,14,18,22-Tetracosahexaene,2,6,10,15,19,23-hexamethyl	21.78	410 (M^+^), 69, 81, 136	3.1

^(a)^ Compounds listed in order of elution from SE30 MS column. ^(b)^ Retention time (in min). ^(c)^ Relative area percentage (peak area relative to total peak area in total ion current (TIC) %).

**Table 3:** Identification of apolar compounds found within the nonpolar fraction of the male cones.

N.	Compound ^(a)^	Rt ^(b)^	Mass Fragmentation (Mass *m*/*z* Values)	RAP ^(c)^
	***n*** **-hexane**			
1	2-Heptenal	6.81	112 (M^+^), 55, 70, 83, 97	0.5
2	Nonanal	9.19	142 (M^+^), 57, 82, 98, 114	0.3
3	2-Decenal	11.00	154 (M^+^), 55, 70, 83, 98, 110	1.1
4	Lauric acid	13.79	200 (M^+^), 43, 60, 73, 129	0.2
5	2-Pentadecanone, 6,10,14-trimethyl	15.57	268 (M^+^), 58, 85, 109, 124, 179	0.2
6	14-Methylpentadecanoic acid	16.06	270 (M^+^), 55, 74, 87, 143	0.9
7	Palmitic acid	16.49	256 (M^+^), 55, 73, 129, 213	1.1
8	Manoyl oxide	16.73	290 (M^+^), 81, 137, 192, 257, 275	0.7
9	1-Octadecene	17.00	252 (M+), 55, 69, 97, 125	0.6
10	9-Octadecenoic acid	17.16	282 (M+), 55, 83, 97, 264	0.8
11	Ethyl linoleate	17.51	308 (M+),55, 67, 81, 95, 157, 263	1.0
12	α-Linolenic acid	17.55	306 (M+), 67, 79, 95, 108, 241, 261	2.2
13	Methyl Palustrate derivate	18.68	314 (M+), 159, 197, 239, 299	3.5
14	Heptadecanoic acid, ethyl ester	18.75	298(M+), 69, 88, 101, 157 129	2.8
15	9-(2-Cyclohexylethyl) heptadecane	19.29	350 (M+), 55, 83, 97, 239	1.7
16	Cyclotetracosane	20.60	336 (M+), 56, 69, 83, 97, 279	3.6
	**Dichloromethane**			
1	1,2-Diphenylcyclobutane	15.09	208 (M^+^), 51, 78, 89, 104	1.3
2	1-propene-1,2,3-tricarboxylic acid,tributyl ester	17.50	342 (M^+^), 57, 84, 112, 139, 157, 213	1.6
3	Palmitic acid β-monoglyceride	19.44	330 (M^+^), 84, 98, 134, 239, 257	1.5

^(a)^ Compounds listed in order of elution from SE30 MS column. ^(b)^ Retention time (in min). ^(c)^ Relative area percentage (peak area relative to total peak area in total ion current (TIC) %).

**Table 5:** Inhibitory effects on nitric oxide (NO) production and cytotoxicity profile.

Sample	Fraction	IC_50_ (µg/mL)	
		NO Inhibition	Cytotoxicity
Female cones	Raw extract	382.00 ± 13.85 ^d^	969.10 ± 10.78 ^f^
	*n*-hexane	107.50 ± 15.22 ^b^	776.90 ± 13.81 ^e^
	CH_2_Cl_2_	226.10 ± 35.77 ^c^	309.10 ± 29.32 ^c^
	AcOEt	n.a.	-
	H_2_O	n.a.	-
Male cones	Raw extract	267.10 ± 22.28 ^c^	-
	*n*-hexane	253.50 ± 23.81 ^c^	285.40 ± 10.59 ^c^
	CH_2_Cl_2_	337.30 ± 35.52 ^d^	336.50 ± 17.28 ^d^
	AcOEt	n.a.	-
	H_2_O	n.a.	-
Indomethacin *		53.00 ± 0.81 ^a^	
L-NAME *		45.86 ± 0.46 ^a^	

Data were expressed as mean ± S.D.M. (*n* = 4). * Positive controls; n.a.= not active. Different letters indicate statistically significant differences at *p* < 0.05 (Bonferroni post hoc test).

**Table 6:** IC_50_ values of inhibition of pancreatic lipase by the extract analyzed.

Sample	Fraction	IC_50_ (mg/mL)
Female cones	Raw extract	0.20 ± 0.01 ^a^
	H_2_O	2.23 ± 0.67 ^b^
Male cones	Raw extract	0.52 ± 0.01 ^a^
	H_2_O	6.30 ± 0.05 ^c^
Orlistat *		0.018 ± 0.001 ^a^

Data were expressed as mean ± S. D. M. (*n* = 3); * Positive control; Different letters indicate statistically significant differences at *p* < 0.05 (Bonferroni post hoc test).

**Table 7:** IC_50_ values of the extracts tested against α-amylase.

Sample	Fraction	IC_50_ (µg/mL)
Female cones	Raw extract	58.54 ± 2.32 ^b^
	n-hexane	n.a.
	CH_2_Cl_2_	n.a.
	AcOEt	121.40 ± 21.22 ^c^
	H_2_O	212.60 ± 27.52 ^d^
Male cones	Raw extract	69.33 ± 7.22 ^b^
	n-hexane	101.00 ± 3.03 ^c^
	CH_2_Cl_2_	35.28 ± 3.08 ^a^
	AcOEt	192.10 ± 6.79 ^d^
	H_2_O	46.77 ± 3.15 ^a^
Acarbose *		12.68 ± 0.54 ^a^

Data expressed as mean ± SEM (*n* = 3); * Positive control; n.a.= not active. Different letters indicate statistically significant differences at *p* < 0.05 (Bonferroni post hoc test).

## Data Availability

The original contributions presented in this study are included in this article/Supplementary Materials. Further inquiries can be directed to the corresponding author.

## Supplementary Materials

The following are available online at https://www.mdpi.com/article/10.3390/antiox14111368/s1, Figure S1: GC-MS Chromatograms of n-hexane and dichloromethane fractions of female and male cones.
