# Supplementary material for: Phytochemical Screening and Biological Activity of Female and Male Cones from Pinus nigra subsp. laricio (Poir.) Maire
Source: Antioxidants (Basel). 2025 Nov 18;14(11):1368. doi: 10.3390/antiox14111368 (PMC12649639; doi:10.3390/antiox14111368)
Supplement: Supplementary file 1 [file antioxidants-14-01368-s001.zip › antioxidants-3954225-supplementary.pdf]

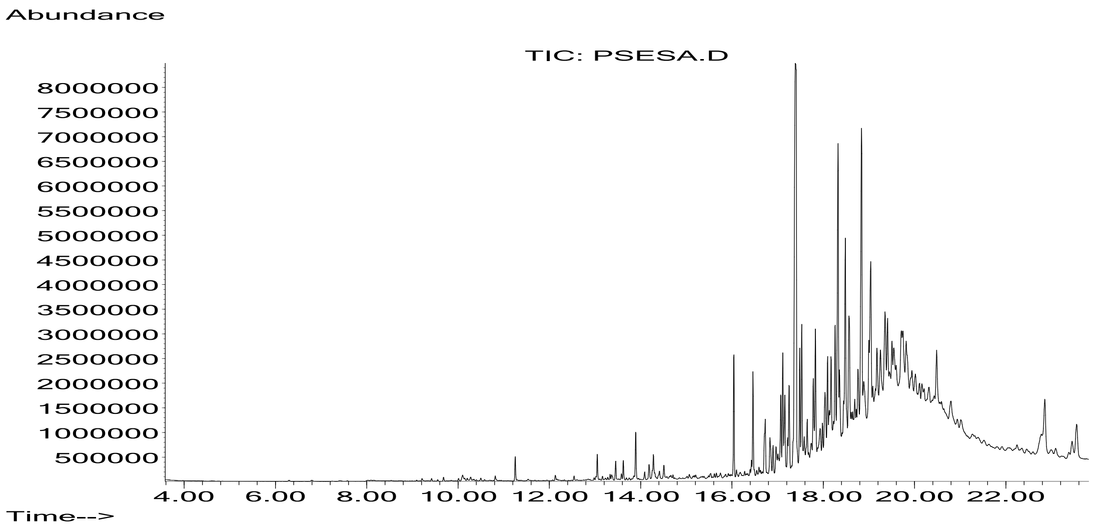

PSESA: FEMALE CONES, HEXANE FRACTION

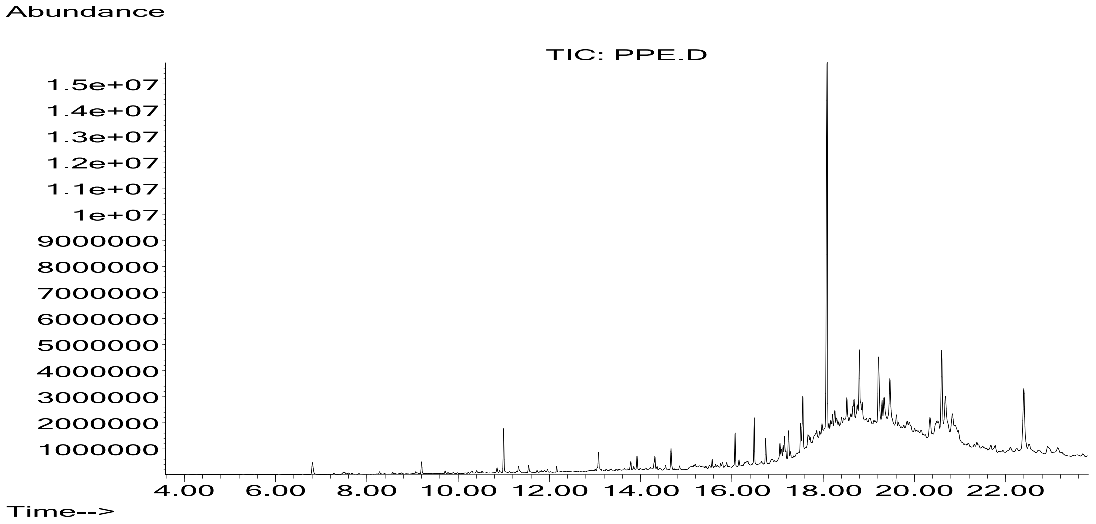

PPE : MALE CONES, HEXANE FRACTION

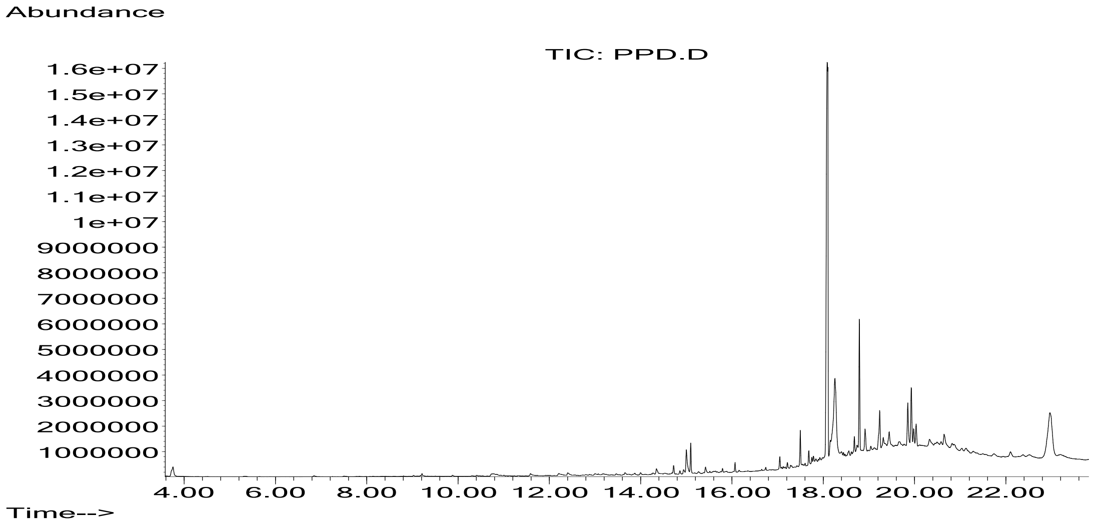

PPD : MALE CONES, DICHLOROMETHANE FRACTION

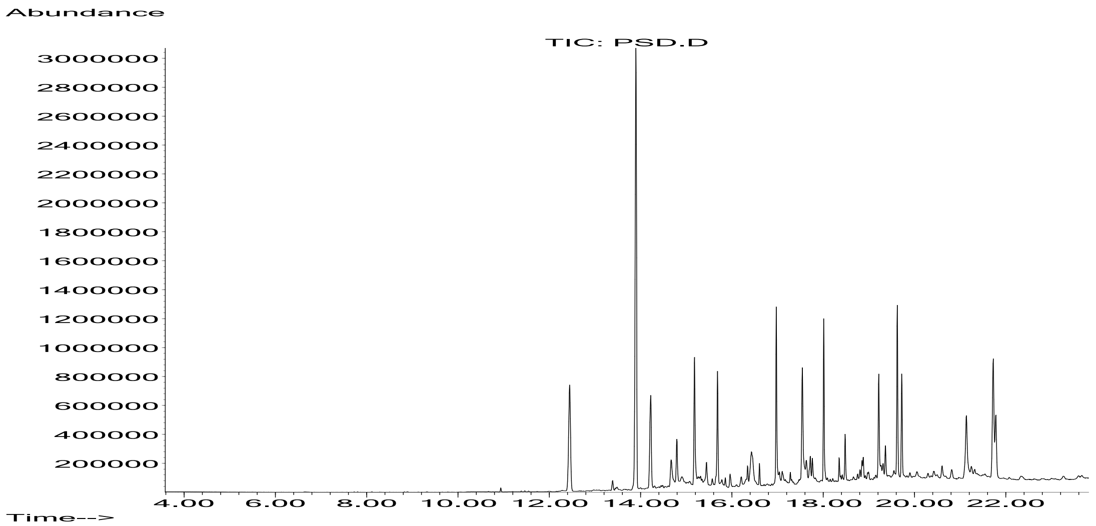

PSD : FEMALE CONES, DICHLOROMETHANE FRACTION
